# Clinical and Angiographic Outcomes After Stent-Assisted Coiling of Cerebral Aneurysms With Laser-Cut and Braided Stents: A Comparative Analysis of the Literatures

**DOI:** 10.3389/fneur.2021.666481

**Published:** 2021-04-29

**Authors:** Longhui Zhang, Xiheng Chen, Linggen Dong, Peng Liu, Luqiong Jia, Yisen Zhang, Ming Lv

**Affiliations:** Department of Interventional Neuroradiology, Beijing Neurosurgical Institute, Beijing Tian Tan Hospital, Capital Medical University, Beijing, China

**Keywords:** intracranial aneurysm, endovascular treatment, stent-assisted coiling, the laser-cut stent, the braided stent

## Abstract

**Introduction:** Stent-assisted coiling (SAC) plays an important role in endovascular treatment of intracranial aneurysms (IAs). This comparative analysis examines the safety and efficacy of SAC in general and compares clinical and angiographic outcomes between laser-cut stents and braided stents.

**Methods:** Relevant English-language studies were identified via a PubMed search for published articles regarding outcomes of SAC using laser-cut stents and braided stents published from 2015 to 2020. Data from 56 studies that met our inclusion criteria were pooled and statistically compared.

**Results:** A total of 4,373 patients harboring with 4,540 IAs were included. Patients were divided into two groups on the basis of stent type: laser-cut stents (2,076 aneurysms in 1991 patients; mean follow-up, 12.99 months) and braided stents (2,464 aneurysms in 2382 patients; mean follow-up, 18.41 months). Overall, the rates of successful stent deployment, thromboembolic events, stent stenosis, periprocedural intracranial hemorrhage, permanent morbidity, mortality, and recanalization were 97.72, 4.72, 2.87, 1.51, 2.14, 1.16, and 6.06%, respectively. Laser-cut stents were associated with a significantly higher rate of successful deployment (*p* = 0.003) and significantly lower rate of periprocedural intracranial hemorrhage (*p* = 0.048). Braided stents were associated with a significantly lower rate of permanent morbidity (*p* = 0.015).

**Conclusion:** SAC of IAs using laser-cut stents or braided stents was effective and safe. Rates of thromboembolic events, stent stenosis, mortality, and recanalization were comparable between the stent types. Braided stents were associated with lower permanent morbidity while laser-cut stents were associated with more favorable rates of successful deployment and periprocedural intracranial hemorrhage.

## Introduction

Surgical clipping and endovascular therapy are the two main treatment options for patients with intracranial aneurysms (IAs). Detection of IAs is increasing because of widespread use of non-invasive intracranial vascular examination techniques such as magnetic resonance angiography. Since the use of Guglielmi detachable coils (GDC) for endovascular IA treatment was introduced in the 1990s (1), the advent of various types of coils and stents has ushered in a Golden Age of endovascular treatment, with stent-assisted coiling (SAC) playing an important role over the past decade.

Patients have widely accepted SAC because of good clinical outcomes (2, 3). In this technique, stent deployment serves as a scaffold to prevent coil prolapse, which preserves the parent artery and promotes thrombosis in the aneurysm and re-endothelialization, resulting in improved outcome (4). Laser-cut and braided stents are the primary intracranial stents in use today. Laser-cut stents are characterized by high vascular compliance, good flexibility, excellent stability, and fewer thromboembolic events in collateral vessels; examples include Neuroform (Stryker Neurovascular, Kalamazoo, MI, USA), Neuroform Atlas (Stryker Neurovascular), Enterprise (Codman Neuro, Raynham, MA, USA), and Solitaire (Medtronic, Dublin, Ireland). Self-expanding braided stents have greater metal surface coverage and provide greater flow diversion to promote aneurysmal occlusion; examples include LVIS (Microvention, Aliso Viejo, CA, USA), LVIS Jr (Microvention), and LEO Baby (Balt, Montmorency, France). Both stent types are widely used in IA management and type superiority has not been established (5, 6).

SAC studies have been increasing owing to a 2013 review of intracranial stenting (7), increased stenting experience, improvements in stenting technology, and increased IA detection; however, results have been variable. Therefore, an up-to-date review of this rapidly developing field is needed. This study aimed to review recent relevant literature to compare clinical and angiographic outcomes of SAC with laser-cut and braided stents and to comment on the safety and efficacy of SAC in general.

## Methods

### Data Sources

The PubMed database was searched for relevant articles published from January 2015 to October 2020 regarding Neuroform, Neuroform Atlas, Enterprise, Solitaire, LVIS, LVIS Jr, and LEO Baby stents. Search terms included “[stent name],” “[stent name] + stent,” and “[stent name] + intracranial aneurysm.” Studies which met the following criteria were reviewed: (a) abstract and/or entire manuscript was published in English; (b) case series, prospective study, or clinical trial that included nine or more patients; (c) detailed clinical and/or radiological post-intracranial stenting data were reported; (d) multiple stent systems were studied with individual system data clearly reported. In cases of controversy, the entire article was thoroughly reviewed. Patients who received more than one type of stent were excluded.

### Data Extraction

The following data from articles meeting our inclusion criteria were reviewed and extracted: sample size, deployment success, thromboembolic events, periprocedural intracranial hemorrhage, stent stenosis, permanent morbidity (present at last follow-up), mortality, angiographic aneurysmal occlusion immediately following the procedure and at last follow-up, and recanalization. Thromboembolic events were defined as stroke, transient ischemic attack, or development of asymptomatic thrombus during the procedure. Stent stenosis was defined as “moderate,” “severe,” or “symptomatic” stenosis, or as ≥50% stenosis if quantified (8–10). Angiographic occlusion was defined in accordance with the Raymond–Roy classification and the modified Raymond–Roy classification (11, 12). Recanalization was defined as “recanalization” or “recurrence”; residual aneurysms that increased in size and aneurysms that progressed to a higher Raymond–Roy class were also considered recanalized. All data were reviewed and verified independently by the two authors and entered into the study database using specialized forms. In some outcome analyses, patients from studies with missing data were excluded from the analysis.

### Statistical Analysis

Statistical analyses were performed using SPSS software version 26 (IBM Corp., Armonk, NY, USA.). Continuous data are presented as means. Categorical data are presented as frequencies or percentages and were compared using the two-tailed Fischer exact test. *P* < 0.05 was considered significant.

## Results

A total of 56 studies comprising 4,373 patients harboring 4,540 aneurysms were included (6, 13–67). Patients were divided into two groups on the basis of stent type: laser-cut stents (2,076 aneurysms in 1991 patients; mean follow-up, 12.99 months) and braided stents (2,464 aneurysms in 2,382 patients; mean follow-up, 18.41 months). Both stents performed equally well, with no significant difference in frequencies of thromboembolic events, stent stenosis events, mortality, recanalization, and class 1 (complete) and class 2 (near-complete) Raymond–Roy angiographic occlusion between groups. Frequency of successful deployment was significantly higher in the laser-cut stent group (98.69 vs. 97.07%, *p* = 0.003), while frequency of periprocedural intracranial hemorrhage was significantly lower (1.13 vs. 1.85%, *p* = 0.048). Frequency of permanent morbidity was significantly lower in the braided stent group (1.58 vs. 2.92%, *p* = 0.015). Rates of clinical complications and class 1 and class 2 angiographic occlusion overall and according to stent type are presented in Table 1. Denominators of the analyses vary based on the number of patients with available data.

**Table 1:** The clinical and angiographic outcomes after stent-assisted coiling of cerebral aneurysms with laser-cut and braided stents.

	**Total *N* (%)**	**Laser engraving stent *N* (%)**	**Braided stents *N* (%)**	***p*-value**
Number of patients	4,373	1,991	2,382	N/A
Number of aneurysms	4,540	2,076	2,464	N/A
Deployment success, %	2957/3026 (97.72)	1202/1218 (98.69)	1755/1808 (97.07)	0.003
Thromboembolic events	210/4451 (4.72)	90/2077 (4.33)	120/2374 (5.05)	0.288
In-stent stenosis	83/2887 (2.87)	32/1089 (2.94)	51/1798 (2.84)	0.909
Peri-procedural intracranial hemorrhage	67/4451 (1.51)	23/2077 (1.13)	44/2374 (1.85)	0.048
Initial Raymond 1	2686/4293 (62.57)	1208/1909 (63.28)	1478/2384 (61.70)	0.392
Initial Raymond 2	1009/4293 (23.50)	441/1909 (23.10)	568/2384 (23.82)	0.587
Initial Raymond 3	598/4293 (13.93)	260/1909 (13.61)	338/2384 (14.18)	0.626
Last follow-up Raymond 1	2453/3109 (78.90)	1175/1478 (79.50)	1278/1631 (78.36)	0.454
Last follow-up Raymond 2	401/3109 (12.90)	175/1478 (11.84)	226/1631 (13.86)	0.097
Last follow-up Raymond 3	255/3109 (8.20)	128/1478 (8.66)	127/1631 (7.79)	0.395
Permanent morbidity	65/3040 (2.14)	37/1268 (2.92)	28/1772 (1.58)	0.015
Mortality	38/3274 (1.16)	16/1316 (1.22)	22/1958 (1.12)	0.868
Recanalization	200/3298 (6.06)	91/1325 (6.87)	109/1973 (5.52)	0.056

*Laser-cut stents: Enterprise, Neuroform, Neuroform atlas, Solitaire*.

*Braided Stents: Lvis, Lvis Jr, Leo baby*.

## Discussion

Our comparative analysis of 4,540 aneurysms in 4,373 patients found that SAC of IAs using either laser-cut or braided stents had favorable clinical outcomes and angiographic results. Overall, rates of mortality, permanent morbidity, and recanalization were low, suggesting that SAC is safe and effective.

Early endovascular coiling studies indicated that low coil density, large aneurysm size, and wide aneurysm neck are recanalization risk factors (68). However, the development of SAC has enabled treatment of wide-neck, complex, and bifurcation intracranial aneurysms. Stent deployment significantly improves outcome by serving as a scaffold to prevent coil prolapse and preserve the parent artery. This promotes thrombosis in the aneurysm lumen as well as re-endothelialization and may explain why SAC has a lower recanalization rate than coil embolization alone. However, stent placement is associated with higher procedural risk and risk of stent thrombosis.

Braided stents in our study were represented by LVIS, LVIS Jr, and LEO Baby. These stents have greater metal surface coverage and provide greater flow diversion, which promotes IA occlusion and results in fewer adverse ischemic events. Laser-cut stents were represented by the Neuroform, Enterprise, and Solitaire series of stents. These stents have the advantages of high vascular compliance, good flexibility, excellent stability, and fewer thromboembolic events in collateral vessels. Our comparative analysis resulted in several significant findings. The rates of successful deployment and periprocedural intracranial hemorrhage favored laser-cut stents; however, permanent morbidity was significantly lower in patients who underwent SAC with braided stents. In addition, rates of angiographic aneurysmal occlusion, thromboembolic events, stent stenosis, mortality, and recanalization did not differ between the two groups. However, several different individual stents were used in each group and the data are representative of multiple device iterations, different operators, and accumulated institutional experience over the past 6 years. Our findings may not be applicable to current practice and should be interpreted with caution.

To be noted, previous studies demonstrated that braided stents have greater metal surface coverage and provide greater flow diversion, which promotes IA occlusion. Feng et al. showed that LVIS stents may achieve lower rate of recanalization and in-stent stenosis than Enterprise (38). Ge et al. reported a higher rate of recanalization associated with enterprise stent than with the LVIS stent (10.7 vs 2.8%) (25). Lim et al. observed in SAC of IAs, laser- cut and braided stent groups produced similar outcomes in follow-up, the difference of recanalization incidence rates between the braided- stent and the laser-cut group was not statistically significant (5).

Unlike previous studies, there are more than one kind of stent in both two groups. Our study finding is similar to that of many previous studies, the recurrence rate of laser-cut stents is higher than braided stents (91/1325 6.87% vs 109/1973 5.52%). Overall, the result was encouraging, with minor difference.

The present studies containing 4,373 aneurysms in more than 4,500 patients in recent 6 years. It is an up-to-date and robust review of this rapidly developing field. This comparative analysis demonstrated that both laser-cut stents and braided stents had good clinical outcomes. Overall, high rate of deployment success (97.72%), initial and final completely and near completely angiographic occlusion was seen in 86.07 and 91.80% of patients, low rate of thromboembolic events (4.72%) and In-stent stenosis (2.87%), low permanent morbidity (2.14%), and mortality (1.16%).

The low clinical complication rate, high deployment success and high degree of occlusion are particularly encouraging, which may result from the increased stenting experience, improvements in stenting technology, and increased IA detection.

The advantages of laser-cut stents characterized by high vascular compliance, good flexibility, excellent stability (24). Besides, the push technique and the release of braided stents as well as catheter pullback are difficult, especially in tortuous vessels. The above mentioned could account for more favorable deployment and less peri-procedural intracranial hemorrhage in laser-cut stents group.

In this study, we made a comparative analysis between the published results of the laser-cut stents and braided stents demonstrated that both laser- cut and braided stent groups produced similar outcomes in SAC of IAs. The pooled data include studies across multiple device iterations in the recent 6 years, the data do not necessarily represent the procedural device failures, clinical complications, or occlusion of the most modern device iterations or procedural techniques used currently. These results should be interpreted with caution.

This study has significant limitations. First, numerous retrospective studies were analyzed, each with their own biases. Second, heterogeneity between the analyzed studies may weaken the generalizability and validity of our findings. Third, the study data were pooled from previously published studies without stringent inclusion criteria, and the scientific quality of the included studies was not graded. Fourth, we did not consider IA location, number of stents used, stent length, stent cost and availability, and different follow-up durations and did not compare stent morphological characteristics between groups, which may have introduced significant bias. Furthermore, direct comparisons between studies were difficult as data reporting was variable. Finally, the reported 6.06% recanalization rate probably significantly underestimates the true rate (69).

## Conclusion

This analysis of 4,373 patients harboring 4,540 IAs who underwent SAC with laser-cut or braided stents within the past 6 years found that SAC is safe and effective. Rates of permanent morbidity, mortality, and recanalization were comparable between the devices; however, braided stents were associated with lower permanent morbidity while laser-cut stents were associated with more favorable rates of successful deployment and periprocedural intracranial hemorrhage.

## Author Contributions

LZ drafted the manuscript and prepared the table. XC, PL, LD, and LJ conducted data collection. ML and YZ conceived and designed this project. All authors reviewed the manuscript.

## Conflict of Interest

The authors declare that the research was conducted in the absence of any commercial or financial relationships that could be construed as a potential conflict of interest.
